# Assessment of the contribution of industrially processed foods to salt and iodine intake in Thailand

**DOI:** 10.1371/journal.pone.0253590

**Published:** 2021-07-06

**Authors:** Saipin Chotivichien, Nuntaya Chongchaithet, Pattamaporn Aksornchu, Nuntachit Boonmongkol, Pattama Duangmusik, Jacky Knowles, Sangsom Sinawat

**Affiliations:** 1 Bureau of Nutrition, Department of Health, Ministry of Public Health, Nonthaburi, Thailand; 2 Iodine Global Network, Seattle, Washington, United States of America; 3 Iodine Global Network, Bangkok, Thailand; 4 College of Allied Health Sciences Suan Sunandha Rajabhat University, Samutsongkhram, Thailand; Università degli Studi di Milano, ITALY

## Abstract

Iodization of food grade salt has been mandated in Thailand since 1994. Currently, processed food consumption is increasing, triggered by higher income, urbanization, and lifestyle changes, which affects the source of salt and potentially iodized salt among the population. However, adequate information about the use of iodized salt in processed foods in Thailand is still lacking. Therefore, this study aimed to assess iodine intake through salt-containing processed foods and condiments which were identified using national survey data. Potential iodine intake from iodized salt in food products was modelled using consumption data and product salt content from food labelling and laboratory analysis. Fish sauce, soy sauce and seasoning sauces (salty condiments) have alternative regulation allowing for direct iodization of the final product, therefore modelling was conducted including and excluding these products. Daily salt intake from household salt and food industry salt (including salty condiments) was estimated to be 2.4 g for children 0–5 years of age, 4.6 g for children 6–12 years of age, and 11.5 g for adults. The use of iodized salt in processed foods (excluding salty condiments) met approximately 100% of the estimated average requirement (EAR) for iodine for non-pregnant adults and for children 6 to 12 years of age, and 50% of the EAR for iodine for children aged 0 to 5 years of age. In all cases, iodine intake from processed food consumption was greater than from estimated household iodized salt consumption. Findings suggest that iodized salt from processed foods is an important source of iodine intake, especially in adults. The use of iodized salt by the food industry should be enforced along with population monitoring to ensure sustainability of optimal iodine intake. Currently, the addition of iodine into fish sauce, soy sauce and seasoning sauces has an important role in achieving and sustaining optimal iodine intake.

## Introduction

Iodine deficiency disorders (IDD) is one of the most significant public health problems in the world. In Thailand, a number of national strategic approaches have been implemented; including legislation for salt iodization, surveillance and monitoring of iodized salt consumption and iodine status, advocacy and social marketing, and research; leading to sustainable elimination of IDD. The salt iodization program has been implemented since 1994 (Ministry of Public Health. Notification No. 153 on Iodized Salt, 18 March 1994. Thailand). According to the notification of Thai FDA, legislation for salt iodization is in place under the Food Act (B.E. 2522) of 1979. The notification of food-grade salt was amended in 2011 to the current legislation which states that “food-grade salt means sodium chloride used in food or used as a mixture or an ingredient in food. Food-grade salt shall contain iodine content not less than 20 milligrams and not more than 40 milligrams per kilogram. Food-grade salt used as mixture or ingredient in food shall be iodized salt” [1]. Moreover, the notification of Thai FDA announced in 2010 stipulated that fish sauce, mixed fish sauce and food seasoning derived from hydrolysis or fermentation of soybean protein, shall have iodine content not less than 2 mg and not more than 3 mg in 1 liter of product, by the direct addition of iodine in the production process or the use of edible iodized salt as an ingredient [2,3].

In 2019, the national production capacity of iodized salt was reported by Thai FDA. The capacity for food-grade iodized salt production was 347,211 tons and food-grade non-iodized salt production was 115,258 tons. Therefore, the ratio between iodized salt and non-iodized food grade salt was 3:1. According to the surveillance and monitoring of national iodine status during 2015–2016 by the Bureau of Nutrition, Department of Health, it was found that household coverage of adequately iodized salt (20–40 mg Iodine/kg) ranged from 78.9% to 79.9% by test kit and from 65.2% to 69.4% using titration method [4]. The 2015–2016 UNICEF-supported Multiple Indicator Cluster Survey (MICS-5) found that, nationally, 73.3% of households with salt were using iodized salt (> 15 mg Iodine/kg), however the methodology used did not permit reliable estimate of the level of iodine in the salt [5]. Moreover, this survey revealed that household iodized salt coverage was different across the regions and by socioeconomic status. Highest household coverage with iodized salt was found in the Southern region, followed by Central, Northern and Bangkok, respectively. The Northeastern region had the lowest coverage [5]. There was also a trend for increasing household use of iodized salt with increasing wealth, households in the highest wealth quintile were most likely to use iodized salt and households in the lowest wealth quintile were the least likely to use iodized salt.

2016 data from the national surveillance of iodine status [4] found adequate iodine status among children 3 to 5 years of age (median urinary iodine concentration (MUIC) 200μg/L, cut off for optimal intake is 100μg/L, n = 3844) and among the elderly (≥ 60 years old) population (MUIC 111μg/L, cut off 100μg/L, n = 3674). However, there was borderline inadequate intake among pregnant women (MUIC 145μg/L, cut off 150μg/L, n = 22,862).

A 2017 national food consumption behavior survey revealed a high frequency of consumption of salt-containing processed foods. The frequency of consumption (at least once in the previous week) of processed meat, ready meals, and snack products was reported among 88.2%, 59.3%, and 48.3% of people aged 6 years and above, respectively [6]. According to the sodium chloride consumption survey, the most frequently used sodium chloride-containing condiments during the previous week were fish sauce (96.4%), food-grade salt (91.5%), soy sauce (64.6%), shrimp paste (63.2%), seasoning powder (61.6%), and oyster sauce (61.4%) for all households. Fish sauce and household salt were each reportedly consumed on average 1–2 times per day for all households [7]. The 2016 food consumption data of Thailand suggested that all age groups consumed salt-containing food products including instant noodles, meatballs, sausages, crab sticks, potato chips, seasoning seaweed, fish snacks, and condiments [8]. According to market research reports, there has been an increase in processed food consumption during the period 2012–2018, including instant noodles, snacks, processed meat products, and condiments [9–12]. Currently, processed food consumption is increasing worldwide, including in Thailand, triggered by higher incomes, urbanization, and lifestyle changes [13–15]. The consumption behavior among the population in Thailand suggests that a wide variety of food, including processed foods are typically consumed. Since Thai legislation includes iodization of both household and food industry salt, the above figures for frequency of food consumption suggest that iodized salt in processed foods may be significant in terms of population iodine intake. Based on these initial findings, our study aimed to investigate the data in more depth and estimate the contribution of iodine intake from iodized salt in widely consumed processed foods and to propose factors that could improve the national iodine strategy.

An additional consideration is the National Salt Reduction Initiative which was initiated by the Thai Bureau of Non-Communicable Diseases (NCDs) in 2016. The goal is to reduce salt intake by 30% by 2025 to reduce the risk of NCDs [16]. Iodine intake from iodized salt would reduce if the national salt reduction policy achieves its goal to reduce salt intake from all sources. Therefore, it was of interest to also investigate the likely effect of successful implementation of this policy on iodine intake from household salt and salt in selected foods.

## Methodology

The project was started in April 2019 and ended in January 2020. In the present study, we, on behalf of the Bureau of Nutrition, Department of Health, collaborated with all relevant stakeholders to consult and provide the data. The national working group and advisory members are listed in the acknowledgements and included partners from different government ministries, academic institutions, and from the food and salt industry.

**Fig 1:**
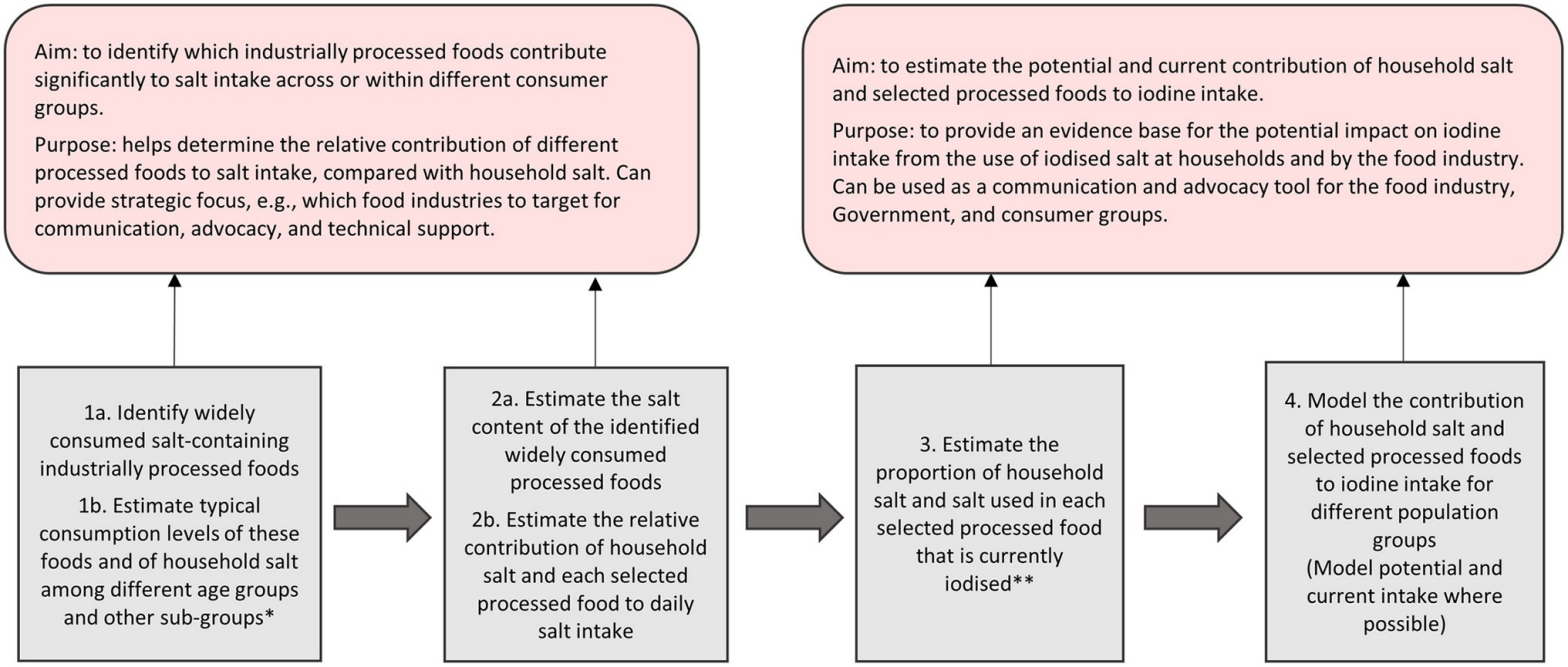
Key guidance framework for Module 4—Assessing the contribution of industrially processed foods to salt and iodine intake. * Population groups included: Children 0–5 years of age, 6–12 years of age and non-pregnant adults. ** The term “Non-pregnant adults” was used in this paper to describe data for the non-pregnant population group above 13 years of age. *** Based on an assumption that, if salt is iodized, it will be iodized to the mean of national standard (30mg/kg).

The study was based on implementing the program guidance on the use of iodized salt in industrially processed foods, developed by The Iodine Global Network (IGN). The main aim of the guidance is to assess iodine intake from iodized salt-containing processed foods and condiments. However, implementation of the 6 modules also guides program managers through a review of iodine nutrition, legislation for salt iodization and its enforcement, and of the enabling environment for effective strategies to achieve and sustain optimal population iodine intake. Background to the guidance and detail about the modules are described in the introductory paper to this collection [see additional materials–*to add reference when the collection is finalised*]. In brief, the methods implemented by the national team followed the 6 modules as follows:

Module 1: reviewed available sources of data related to typical intake of widely consumed industrially processed foods, the salt content of identified food, and iodized salt use by households, as cooking or table salt, and by the food industry. These sources included A) a national food consumption behavior survey using stratified two-stage sampling to demonstrate the frequency of consumption of processed meat, ready meal, and snacks products among the population 6 years of age and above [6]. B) A national sodium chloride intake survey which investigated intake of the main food and condiment sources of sodium chloride for all age groups, as well as the salt content of these products using 7-day food list recall and 3 day weighted inventory methods [7]. C) Food consumption data of Thailand which estimated consumption for a wide range of foods using semi-quantitative methods, with 24-hour dietary recall among a sub-group of the sampled population for all age groups [8]. D) A series of market research reports which reported on trends and company shares of industrially processed foods including instant noodles, processed meats, snacks, and condiments [9–12]. And E) reports from the Thai FDA on the quantity of food-grade salt and iodized salt production for domestic use [4], as mentioned in the introduction to this manuscript.
Module 2: reviewed current knowledge of the national iodine situation, including household coverage of adequately iodized salt, iodine status among different population groups, details of these are provided in the introduction.
Module 3: reviewed the national legislation on salt iodization and enforcement, including its application to salt-containing food and condiments, as described in the introduction.
Module 4: assessed the potential and estimated current iodine intake from the iodized salt in household salt and in salt-containing processed foods and condiments, based on consumption data. A framework outlining the steps taken to make this assessment is shown in Fig 1. and explained in more detail in the introductory paper to this collection. [see additional materials–*to add reference when the collection is finalised*]. Additional details of this assessment in the Thai context are included below.
Module 5: reviewed the strengthens and weaknesses of national strategies related to the use of food-grade iodized salt.
Modules 6: recommended areas for strategic change/strengthening according to the study findings, for policy makers and food manufacturers, with the aim to ensure optimal iodine nutrition among the population.

### Assessment of the salt and iodine intake from selected processed foods and condiments

In this investigation, reference the salt-containing processed foods included both processed foods and condiments. Widely consumed salt-containing processed foods were selected for inclusion based on a review of national surveys [6–8] and market research reports [9–12], as described for Module 1 above.

The salt content of the selected salt-containing processed foods was estimated based on food product labelling and laboratory analysis. The food consumption surveys did not collect information on processed food brands, however the estimation of product salt content for the identified processed foods was based on an average salt content from at least 3 brands with high market share brands per product. Market share for different brands was identified through the review of market share reports [9–12].

The estimation of average daily per capita salt intake from selected food items was done by multiplying the percent dry weight salt content by the estimated daily consumption quantity in grams. For one food product this was calculated as:

$$Estimated\;daily\;salt\;intake\;from\;each\;product\;\left(g\right)=Estimated\;average\;daily\;per\;capita\;consumption\;of\;each\;product\;\left(g\right)\;x\;product\;salt\;content\;\left(\%\;product\;weight\right)$$ (1)

Iodine intake from iodized household and food industry salt was modelled for five different scenarios, as summarized in Table 1.

**Table 1:** The different scenarios used to model iodine intake from iodized salt in household salt and selected industrially processed foods.

Scenario	Situation represented by the Scenario	Modelling of intake of iodine from iodized salt from household salt and selected processed food products based on iodization at 30mg/kg with up to 30% loss of iodine in final product			
		Household salt	Food industry salt	Iodization of fish sauce, soy sauce and seasoning sauce	Percent salt reduction across all products
**1**	Universal Salt Iodization	100% iodized	100% iodized	Included/Excluded	0%
**2**	Current national situation	78% iodized	100% iodized	Included/Excluded	0%
**3**	National target situation	90% iodized	100% iodized	Included/Excluded	0%
**4**	Current national situation with salt reduction	78% iodized	100% iodized	Included/Excluded	30%
**5**	National target situation with salt reduction	90% iodized	100% iodized	Included/Excluded	30%

Scenario 1 illustrates potential iodine intake, if 100% of food grade salt (household salt and food industry salt) was iodized. This was calculated for household salt and each processed food product, using Eq (2) below. Multiplying expected iodine intake by 70% was included to account for a possible 30% loss of salt iodine from production of the salt to the time of consumption.


$$Potential\;iodine\;intake\;from\;daily\;intake\;of\;each\;product\;\left(\mu g\right)\;for\;Scenario\;1=Estimated\;daily\;salt\;intake\;from\;each\;product\;\left(g\right)\;(see\;equation\;\left(1\right))\;x\;national\;iodized\;salt\;standard\;\left(30\;mg/kg\right)\;x\;70\%$$ (2)

All modelled Scenarios (1 to 5) included the current estimate of 100% iodization of food industry salt. Scenario 2 and 3 were therefore based on the same Eq (2) for iodine intake from processed food salt, however, iodine intake from household salt was multiplied by the current estimated percent of households using iodized salt (78% for Scenario 2) and by the national target percent for households using iodized salt (90% for Scenario 3). Eq (3) shows the calculation for Scenario 2 and 3.

$$Potential\;iodine\;intake\;from\;daily\;intake\;of\;household\;salt\;\left(\mu g\right)=Estimated\;daily\;intake\;of\;household\;salt\;\left(g\right)\;(see\;equation\;\left(1\right))\;x\;national\;iodized\;salt\;standard\;\left(30\;mg/kg\right)\;x\;70\%\;x\;a$$ (3)

*whereas* “*a*” *equal to 78% (for scenario 2) or 90% (for scenario 3)*

Scenario 4 and 5 included a factor to account for the salt reduction target of 30% for intake of household salt and of salt in all processed food products, as shown in Eq (4) for Scenario 4.

$$Potential\;iodine\;intake\;from\;daily\;intake\;of\;household\;salt\;\left(\mu g\right)=Estimated\;daily\;intake\;of\;household\;salt\;\left(g\right)\;(see\;equation\;\left(1\right))\;x\;national\;iodized\;salt\;standard\;\left(30\;mg/kg\right)\;x\;70\%\;x\;a\;x\;70\%$$ (4)

*whereas* “*a*” *equal to 78% (for scenario 4) or 90% (for scenario 5)*

Modelling for each of the five Scenarios was conducted including and excluding the use of iodized salt in fish sauce, soy sauce, and seasoning sauce because national legislation allows for iodization of these specific products using iodized salt or through the direct addition of iodine (2–3 mg/L). In practice, iodization is usually achieved through direct iodization so the expected intake of iodine from these sauces would be different (explained in the discussion section).

Estimates for potential and estimated current iodine intake (μg) from household salt and from the selected processed foods were presented as a percentage of the age-appropriate estimated average requirement (EAR), recommended nutrient intake (RNI) and tolerable upper intake limit (UL) for iodine. The dietary reference values used for these three metrics are shown in Table 2.

**Table 2:** EAR, RNI and UL for iodine (μg) in different age groups [17].

Population group (years of age)	EAR	RNI	UL
	Average daily intake for healthy individuals in a particular life stage and age groups		
	Estimated to meet requirements of 50% population	Estimated to meet the requirements of 97.5% population	Highest intake likely to pose no risk of adverse health effects to almost all individuals
Children (0–5)	65	90	Not available
Children (6–12)	65	120	300
Non-pregnant adults (over 13)	95	150	600

EAR: Estimated average requirement, RNI: Recommended nutrient intake, UL: Tolerable upper intake, for iodine.

Meetings of the national working team were held in August and October 2019. The meetings focused on the modelled scenarios of iodine intake from processed foods and on future monitoring of the use iodized salt in processed foods.

A consultation meeting between the national working and the advisory teams was hosted by the Bureau of Nutrition of Department of Health in October 2019. The participants were the Institute of Nutrition of Mahidol University, and Thai Food and Drug Authority (FDA). The meeting focused on interpretation of the assessment results in relation to decisions on continued addition of iodine to fish sauce and fermented soybean products.

## Results

### The consumption of salt-containing processed foods and condiments

The data collection from the national surveys [6–8] and market share reports [9–12] suggested that household salt and 26 industrially-processed food products contributed significantly to salt intake among different age groups (Table 3). The industrially processed foods and condiments contained food grade salt in a range between 0.6% (potato chips) to 86.3% (seasoning powder with high salt content) of product weight. Instant noodles, meat products (meatballs, sausage), and processed seafood were all products with relatively high volume of consumption (> 5 g/day) in all population groups, however, they had a salt content by weight of < 5%. While condiments had salt content by weight, ranging from 3% to 31%, they were generally consumed in smaller amounts (< 4 g/day where data were available, except for fish sauce consumption among adults which was estimated at 11.6 g/day).

**Table 3:** The average product salt content and consumption of salt-containing processed foods and condiments.

Food products	Average salt content (% product weight)	Average daily food consumption among different population groups (per capita; g/day)		
		Children (0–5 yrs old)	Children (6–12 yrs old)	Non-pregnant adults
Household salt	100	0.4	0.6	3.1
Seasoning powder (high salt content)	86.3	No data	No data	1.3
Seasoning powder (medium salt content)	42.8	No data	No data	1.3
Fish sauce	30.6	1.4	2.6	11.6
Shrimp paste	17.6	No data	0.5	2.9
Seasoning powder (low salt content)	15.1	No data	No data	1.3
Soy sauce	13.1	0.9	1.2	3.2
Fermented fish sauce	11.3	0.5	1.2	2.3
Seasoning sauce	10.9	0.9	1.5	1.6
Oyster sauce	7.9	No data	No data	2.2
Black soy sauce	5.8	No data	No data	1.0
Chili sauce	5.8	No data	1.2	1.1
Instant noodles	4.8	6.5	11.8	7.7
Sweet chili sauce for chicken	4.5	0.3	0.9	0.7
Meatball (pork, chicken, beef))	4.4	8.3	22.4	18.1
Ketchup	3.1	0.9	1.7	1.0
Suki Sauce	3.0	0.3	0.8	1.1
Sausage (sausage, bologna, ham)	2.7	10.9	13.5	7.5
Canned fish	1.9	1.4	4.6	3.3
Processed seafood (crab stick, fish ball, shrimp ball)	1.7	5.8	10.6	5.9
Wheat snack	1.7	2.1	3.3	1.5
Seasoned seaweed	1.5	0.8	1.4	0.6
Popcorn/corn snack	1.5	2.3	4.2	No data
Rice/tapioca snack	1.4	2	5.4	2.2
Nuts	1.1	2.4	3.3	2.8
Fish snack	1.0	0.6	1.5	0.9
Potato snack	0.9	1.6	1.9	1.0
Potato chips	0.6	3.4	6.2	4.0

### Estimation of daily salt and potential iodine intake among different population groups

The estimated daily salt intake from household salt together with salt from identified processed foods for each group was 2g for children 0–5 years of age (including 20 processed food products), 5g for children 6–12 years of age (including 22 processed food products), and 12g for non-pregnant adults (including 26 processed food products). Processed foods included fish sauce, soy sauce and seasoning sauce for all groups, as shown in Table 4. We evaluated the potential iodine intake using current estimates of 78% of household using iodized salt and 100% of food industry salt being iodized (Scenario 2), including iodized salt for fish sauce, soy sauce and seasoning sauce; then used this to assess the potential contribution to the EAR, RNI and UL for iodine among the different population groups from these selected processed foods and household salt, also shown in Table 4.

**Table 4:** The assessment of average daily iodine intake for Scenario 2 (including fish sauce, soy sauce and seasoning sauce).

Population groups	Estimated daily salt intake from household salt and the selected processed foods (g)	Potential iodine intake from household salt and salt in the selected processed foods (μg)	Potential percent contribution to iodine EAR, RNI and UL from household salt and salt in the selected processed foods based on 78% iodization of household and 100% of food industry salt		
			% EAR	% RNI	% UL
Children 0–5 years of age (20 PF)	2	49	75%	54%	No UL reference available
Children 6–12 years of age (22 PF)	5	94	145%	78%	31%
Non-pregnant adults over 13 years of age (26 PF)	12	230	242%	153%	38%

PF: Processed foods, EAR: Estimated average requirement, RNI: Recommended nutrient intake, UL: Tolerable upper intake limit; for iodine.

The contribution to iodine EAR for the different population groups using scenario 2 ranged from 75% among the youngest age group, to 242% among adults. The contribution to the RNI for iodine for children 0–5 years, children 6–12 years, and non-pregnant adults was 54%, 78%, 153%, respectively. Whereas the contribution to the UL for iodine was 31% among children 6–12 years old and 38% among adults. There is no established UL for children 0–5 years of age.

### Models of estimated iodine intake from household salt and selected salt-containing processed foods for the Scenarios 1 to 5

**Fig 2:**
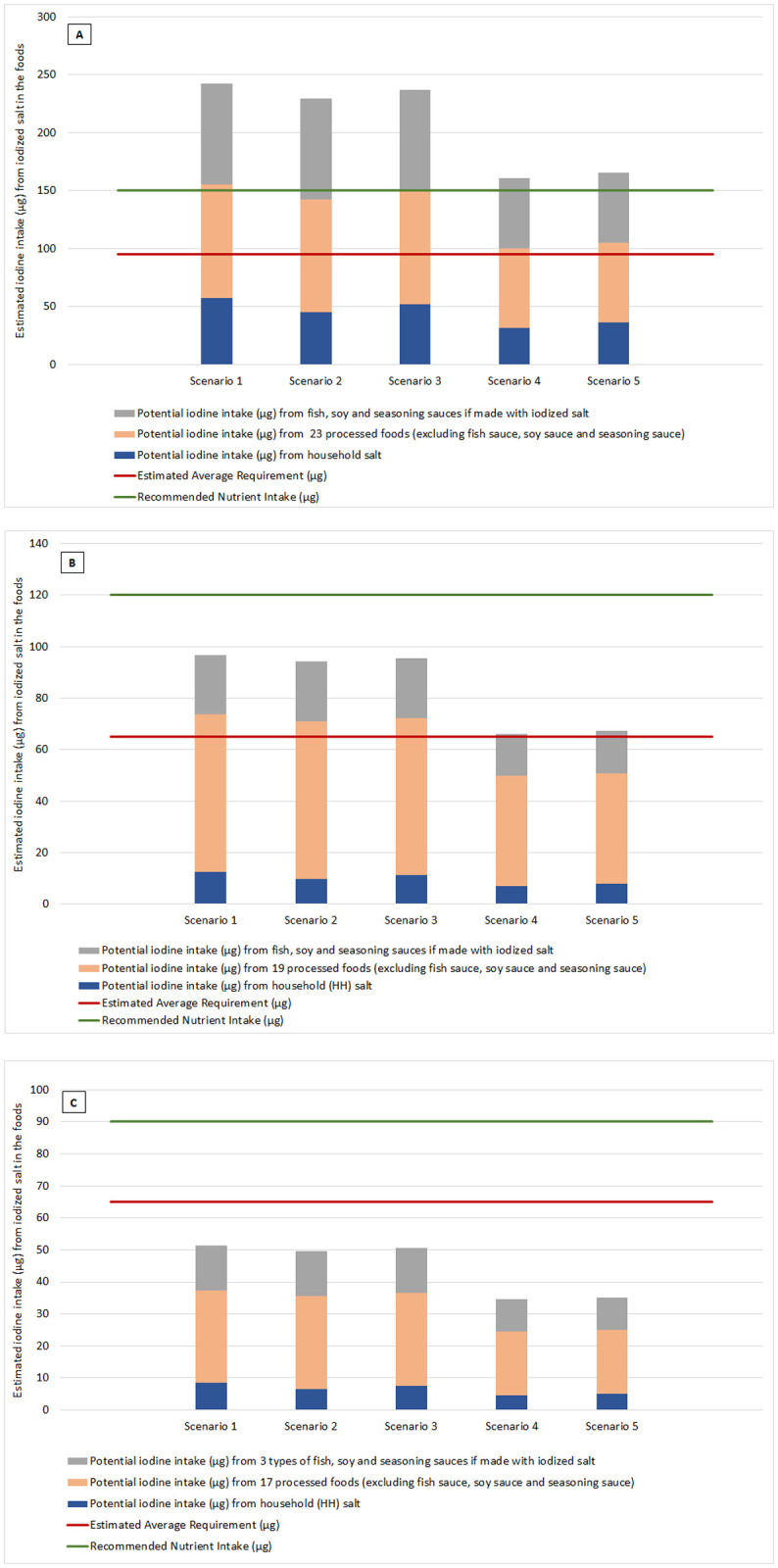
Estimated iodine intake (μg) from the five different scenarios, modelled to show the different contribution from iodization of household salt, processed food salt, and salt in fish sauce, soy sauce and seasoning sauce, in relation to the EAR, RNI and UL for iodine. (A) Non-pregnant adults, (B) Children 6–12 years of age, (C) Children 0–5 years of age. Scenarios: (1)– 100% iodized household and food industry salt, (2)– 78% household salt iodized, 100% food industry salt iodized, (3)– 90% household salt iodized, 100% food industry salt iodized, (4)–Scenario 2 with 30% reduction in salt consumption from all products, (5)–Scenario 3 with 30% reduction in salt consumption from all products.

Iodine intake based on the five different scenarios was estimated using the typical food intake data shown in Table 3, the current estimate for iodization of household salt (78% iodized), the national target for household salt iodization (90% iodized) and best available information regarding iodization of food industry salt (100% iodized). The outcome from modelling iodine intake for each scenario is shown in Fig 2A (for non-pregnant adults), 2B (for children 6–12 years of age) and 2C (for children 0–5 years of age). The charts indicate the expected iodine intake from different levels of household salt iodization described for each scenario and 100% iodization of salt in the selected processed foods. The bars for each scenario are divided to show the relative iodine intake from household salt; processed foods not including fish sauce, soy sauce and seasoning sauces; and from fish sauce, soy sauce and seasoning sauce. Estimated iodine intake is represented in relation to the EAR, RNI and for iodine for each age group, where these reference values are available.

Results for adults (Fig 2A) suggest that iodine intake from current or target iodization of household salt (78% or 90% iodized–Scenarios 2 and 3) together with iodized food industry salt (excluding fish sauce, soy sauce, and seasoning sauces) is sufficient to meet the EAR and RNI. Meanwhile, the iodine intake from processed foods without inclusion of iodine from household iodized salt, fish sauce, soy sauce and seasoning sauces is less than the RNI. Typical levels of household salt consumption alone meet 47% to 55% of the EAR and 30% to 35% of the RNI for iodine in both scenario 2 and 3.

If the 30% salt reduction goal is achieved across all products, the adult intake of iodine from iodized processed food and household salt excluding fish sauce, soy sauce and seasoning sauces, would be approximately equal to the EAR but would not meet the RNI. This is shown in Fig 2A. by the salt reduction scenarios: Scenario 4 (78% household salt iodized) and Scenario 5 (90% household salt iodized). Iodine intake could meet the RNI for iodine if iodized salt was used in fish sauce, soy sauce and seasoning sauces. In all five scenarios, iodine intake is well below the tolerable upper limit of 600μg.

Results for children aged 6–12 years (Fig 2B) suggest that iodine intake from iodized household salt (78% or 90% iodized–Scenarios 2 and 3) together with iodized food industry salt is sufficient to meet the EAR without inclusion of iodine from fish sauce, soy sauce and seasoning sauces. However, just over half the RNI for iodine is met by these same two scenarios. Even if fish sauce, soy sauce and seasoning sauce were made with iodized salt, the modelling for scenarios 2 and 3 indicates it is unlikely that the RNI for iodine for children 6 to 12 years of age would be met from all 22 selected food products and household salt combined. If the 30% salt reduction goal is achieved across all products, the intake of iodine from these salt sources would reduce to a level lower than the EAR for children 6–12 years of age (Scenarios 4 and 5 of Fig 2B), even if fish sauce, soy sauce and seasoning sauces included iodized salt. In all five scenarios, iodine intake is less than half the tolerable upper limit of 300μg.

Results for children aged 0–5 years (Fig 2C) suggest that iodine intake from iodized household salt (78% or 90% iodized–Scenarios 2 and 3) together with iodized food industry salt in 17 processed food products is not sufficient to meet the EAR or RNI, with or without inclusion of household salt, fish sauce, soy sauce and seasoning sauce. If the 30% salt reduction goal is achieved across all products, the intake of iodine from all these salt sources would reduce to a level approximately only half the EAR, as shown by Scenarios 4 and 5. No tolerable upper limit for iodine intake has been established for this age group.

Table 5 shows estimated iodine intake by processed food product, for the 12 processed foods contributing most to iodized salt intake. Data are based on an assumption of 100% salt iodized to the mean of the national standard, 30% loss of iodine in the product at time of consumption, and typical daily adult consumption quantities (Table 3). Data indicate that the high daily intake of fish sauce and its high product salt content, means that, if produced with iodized salt, it has the potential to contribute to 183% of the EAR and 116% of the RNI for iodine among non-pregnant adults. Taken together, typical daily consumption of fish sauce, seasoning powder (high and medium salt content), meatballs, shrimp paste, soy sauce and instant noodles, have the potential to meet both the EAR and RNI for iodine among adults if all these processed foods are made with iodized salt. Without iodine from iodized salt in fish sauce, soy sauce, and seasoning sauce; the remaining 9 processed foods are likely to contribute to around 92% of the EAR and about 58% of the RNI for iodine for non-pregnant adults. In practice, the direct iodine addition in fish sauce, soy sauce and seasoning sauce (at 2–3 mg/L) together with iodized salt in processed foods would contribute to an estimated iodine intake meeting 136% of EAR and 86% of RNI.

**Table 5:** Estimated iodine intake (μg) from iodized salt in the 12 processed foods contributing the most to daily iodine intake (based on iodized salt except for fish sauce, soy sauce and seasoning sauce).

Food type	Current estimated daily iodine intake (μg) from iodized salt in each product		
	100% iodized salt used in all 12 products	100% iodized salt used in 9 products, not in fish sauce, soy sauce and seasoning sauce	100% iodized salt used in 9 products, with direct addition of iodine (2–3 ppm) for fish sauce, soy sauce and seasoning sauce
Fish sauce	74	0	29
Seasoning powder (high salt)	23	23	23
Meat ball (Pork, chicken, and beef)	17	17	17
Shrimp paste	11	11	11
Seasoning powder (medium salt)	11	11	11
Soy sauce	9	0	8
Instant noodles	8	8	8
Fermented fish sauce	6	6	6
Sausage (Sausage, bologna, ham)	4	4	4
Seasoning powder (low salt)	4	4	4
Seasoning sauce	4	0	4
Oyster sauce	4	4	4
**Total estimated daily iodine intake (μg) from iodized salt or direct iodization of selected foods**	**175**	**88**	**129**

Scenario 2 for non-pregnant adults.

## Discussion

The assessment described in this paper indicates that the estimated current iodine intake from the use of iodized household salt (78%) and 100% iodized salt in industrially processed foods (Scenario 2), is sufficient to meet the iodine requirements of the majority of the non-pregnant adult population (indicated by the RNI). This is true when fish sauce, soy sauce and seasoning sauces are made with iodized salt (iodine intake well above the RNI) or with non-iodized salt (borderline RNI for iodine achieved). There are no recent data for iodine status among adults over 18 years of age, however, status among older adults (≥ 60 years old) appears sufficient and the status of pregnant women is borderline sufficient [4], which appears to agree with the results of this assessment on the adequacy of likely iodine intake.

The data for children present a different picture. Without iodine from fish sauce, soy sauce and seasoning sauces, estimated iodine intake from household salt and other processed foods provides adequate iodine to meet the requirements of 50% of a healthy population of children 6 to 12 years of age (the EAR) but is below the level of intake required to meet the needs of almost all the population (the RNI). Among younger children, 0 to 5 years of age, iodine intake from foods included in this assessment is insufficient to meet even the EAR.

Given that iodine status among children under 6 has been shown to be adequate [4], the results of the assessment indicate that there are likely to be other sources of iodine in the diet, possibly iodized salt in processed foods not included in the assessment or from iodine in eggs, meat and dairy products, as a result of iodine supplementation of poultry and animal feed. In support of this, the 2016 Thai food consumption data, reported that children 0–5 and 6–12 years of age typically consume 148ml and 86ml of milk a day, respectively [8]. Analysis of the iodine content of cow’s milk indicates that these levels of milk of intake would provide approximately 44 μg and 26 μg of iodine, respectively [18]. This is equivalent to 49% and 22% of the respective age related RNI values for iodine. Iodized salt can be regarded as an essential source of iodine to ensure adequacy of intake when combined with these other non-salt sources, which are likely to be more variable in their daily intake and iodine content. Unfortunately, no food consumption data are available for children 0–1 years of age. However, the investigation of urinary iodine content among breastfed infants (4–6 months old) found that the median urinary iodine of breastfed infants was greater than for formula-fed infants, it is assumed that the status of breastfed infants reflects maternal practices regarding consumption of iodine-containing foods [19].

The assessment was conducted to obtain an evidence base from which to review the existing national salt iodization and salt reduction strategies in order to successfully sustain adequate iodine intake. A key strategic consideration was whether iodine from using iodized salt or from direct iodization of fish sauce, soy sauce and seasoning sauces contributed to maintaining optimal iodine intake. The data for children indicate that iodization of these products is important to overall sufficiency of intake and will become even more important if the salt reduction target is achieved, resulting in a concurrent decrease in iodine intake. The iodine level of iodized salt or the level added to these salty condiments could be adjusted where evidence shows this is needed.

The assessments were conducted based on inclusion/exclusion of iodized salt when compared with non-iodized salt in fish sauce, soy sauce and seasoning sauce. The alternative permissible industry practice for these products is to add iodine directly to the final product. If all these products were iodized to achieve 2.5mg iodine/liter in the final product, iodine intake based on typical daily consumption for adults, could be approximately 50% of the iodine intake indicated in the results in this paper (based on the use of iodized salt).

Building on all the above findings, most importantly current adequacy of population iodine status, the national working and advisory team consultation meeting recommended to maintain the current standard salt iodine level at 20–40 mg/kg. This could be considered for revision if future successful salt reduction indicates that the associated decrease in iodine intake results in inadequate population iodine intake. At the same time, regular campaigned to increase consumer awareness about the benefit of lowering salt intake to prevent hypertension will be combined with messaging about the use of iodized salt. The findings in this paper report typical salt consumption among adults in Thailand is more than double the WHO recommended intake of 5g/day. This is a well-documented risk factor for non-communicable-disease [20], therefore the national salt reduction strategy is timely and important WHO guidance confirms that salt iodization and salt reduction strategies are highly compatible and should be implemented and monitored collaboratively to ensure maintenance of optimal iodine intake alongside achieving the public health goal of reduced salt intake [21,22].

Moreover, the group suggested to continue the current requirement of adding iodine into fish sauce and fermented soy products until and unless there is more information to confirm that the population in Thailand would not suffer from iodine deficiency without the additional iodine from these products. Withdrawal of the requirement had been considered due to the difficulties reported with quality control of the iodine level from direct addition of potassium iodide into the final products before bottling. Furthermore, regulatory monitoring of the iodine content in fish, fermented soybean and seasoning sauces has been conducted nationally using two methods, spectrophotometer and inductive coupled plasma techniques, which do not produce comparable results. Therefore, all members recommended that the Thai FDA considers how to strengthen compliance and control of the iodine content of these products.

Other recommendations from the consultation meeting were to introduce regulatory monitoring protocols for the food industry, to control and monitor the use of iodized salt during food processing. Communication and engagement with the food industry are considered as important factors to facilitate this. The quality of iodized salt production in the range of 20–40 mg/kg also needs to be assured through continued regulatory monitoring of salt production and imports. The national working group also recommended to strengthen urinary iodine monitoring among different groups, as important to assure sustained optimal iodine nutrition, in particular as salt reduction interventions are implemented, and to research other factors that might affect iodine intake among different population groups.

Raising public awareness about the adverse effects of iodine deficiency through public relations and social marketing campaigns are important and are already a part of the Thai national strategy.

Limitations to the study conducted include the following. The application of broad food intake estimates to the whole population in a given age group, although there is known to be a reasonable amount of regional variation in the types of foods and level of consumption. Also, the application of 30% salt reduction across all products, including household salt enabled an illustration of the need to implement and monitor salt iodization and salt reduction strategies together. However, in reality, some food products would be more appropriate for reformulation with reduced salt content than others, and a reduction of 30% in household salt consumption would require substantial behavior change, which may not be as achievable as reformulation of some processed foods.

Future plans are to model the likely impact on iodine intake from product specific adjustment to salt reduction targets, as these are developed, and to conduct the same modelling by region and socioeconomic status, to the extent that is possible based on available food consumption data in Thailand.

## Conclusion

The findings helped the national program management team to recognize that processed food is an important additional source of iodine to iodized household salt. A greater focus on both components will help ensure that the whole population benefits from this source of iodine intake regardless of dietary choices and practices related to the use of household salt and consumption of processed foods. The different scenarios for iodine intake from processed foods assisted the team to understand the current sources of iodine, how these could change and the likely impact in regard to sustainable prevention of iodine deficiency.

The outcomes from the assessment led to national strategy recommendations to monitor the use of iodized salt in processed foods. Therefore, the results are expected to strengthen the national program for achieving and sustaining optimal iodine nutrition. It was useful to identify gaps in information related to iodine intake among the population in Thailand.

A key strategic recommendation was that monitoring the use of iodized salt by the food industry should be brought under the national salt iodization strategic plan to ensure sustainability of optimal population iodine intake. Furthermore, the addition of iodine into fish sauce, soy sauce and seasoning sauces has an important role in achieving and sustaining optimal iodine intake.

## Supporting information

S1 File Estimated iodine intake in 0-5years. (XLSX)

S2 File Estimated iodine intake in 6-12years. (XLSX)

S3 File Estimated iodine intake in non-pregnant adults. (XLSX)

## Abbreviations

EAR: estimated average requirement
IDD: iodine deficiency disorders
IGN: Iodine Global Network
MICS: Multiple Indicator Cluster Survey
MUIC: median urinary iodine concentration
NCDs: non-communicable diseases
PF: processed foods
RNI: recommended nutrient intake
UL: tolerable upper intake limit
